# Abdominal Wall Dyskinesia in a Child Presenting as Belly Dancers’ Syndrome: A Case Report

**DOI:** 10.31729/jnma.8296

**Published:** 2023-10-31

**Authors:** Divya KC, Ajaya Kumar Dhakal, Devendra Shrestha, Sharda Acharya, Nischal Neupane

**Affiliations:** 1 Department of Pediatrics, KIST Medical College and Teaching Hospital, Imadol, Lalitpur, Nepal

**Keywords:** *case reports*, *dyskinesias*, *salbutamol*

## Abstract

Belly dancer's dyskinesia or syndrome is a rare condition characterized by involuntary, undulating, infrequent diaphragm movements. The etiologies for this disorder include nervous system disorders (peripheral or central), drug-induced, psychological, or idiopathic. This article describes a 10-year-old boy with an underlying psychological stressor who suddenly experienced involuntary abdominal wall movements after salbutamol nebulization. The child was diagnosed with salbutamol-induced belly Dancer's Dyskinesia with an underlying psychosocial issue following a thorough history, medical examination, and abdominal ultrasonography demonstrating fast rhythmic diaphragm movements. These movements subsided with medical and psychological therapy for two weeks. Belly dancer's dyskinesia is a complex disorder that is difficult to diagnose but can be managed with medical treatment and psychological counseling alone in a few patients. In contrast, in other cases, surgical intervention may be required.

## INTRODUCTION

Belly dancers dyskinesia (BDD) is a rare condition involving myoclonic diaphragm movements. The name BBD was given due to its resemblance to the movements of a belly dancer, first termed as diaphragmatic flutter in 1723.^1^ This rare condition is now known as BDD due to its resemblance to the movements of a belly dancer. This disease's pathophysiological basis or etiology has yet to be concretely described. Few case report illustrated the connections between BDD and conditions such as central nervous system disorders and drugs (salbutamol).^2^ There is no specific diagnostic tool or procedure to diagnose BDD, although diaphragmatic electromyography or ultrasound fluoroscopy are supportive diagnostic tests.^3^ Treatments are primarily aimed at the cause or are mostly symptomatic.

## CASE REPORT

A 10-year-old male presented to the Pediatric Emergency with complaints of sudden onset of frequent, intermittent, involuntary abdominal wall jerky movements that were non-progressive. It was associated with sharp pain over the left chest for 4 hrs before the presentation. The child was receiving salbutamol nebulization for treatment of acute exacerbation of bronchial asthma (unknown dose) 2 days prior to admission. The abdominal wall movements started after 1 hr of salbutamol nebulization, each episode lasting 3-5 min. These movements were persistent while the child was distracted. There was no history of seizures, limb tingling, difficulty walking, drug intake, palpitations, fast breathing, trauma, or falls. The child received immunization as per Nepal's national immunization schedule. The child has been going to school regularly, but recently there has been a slight deterioration in class performance. There was no history of similar illnesses of myoclonus among family members.

The child was alert, active, and interactive during clinical examinations. His saturation was normal, with no signs of respiratory distress, and pulse rate, respiratory rate, and blood pressure were normal for his age. However, he was underweight (BMI 12.9 kg/m^2^). On systemic examination, wheeze was auscultated in bilateral auscultatory areas. The cardiovascular examination was normal. Gait, power, tone of the muscles, reflex on all four limbs, and cervical-spinal movements were normal. Hepatomegaly, splenomegaly, and lymphadenopathy were absent. The child was admitted to the Pediatric ward to monitor movements. These rhythmic movements disappeared during sleep.

Complete blood count, liver function test, renal function test, electrolytes (sodium, potassium, and calcium), chest x-ray, and electrocardiogram (ECG) were normal. Ultrasound abdomen showed a rhythmic and regular contraction of both diaphragms during dyskinetic movements, while the rest of the scan was unremarkable. At the same time, the rest of the scan was unremarkable. MRI head and echocardiography were also normal. Due to the unavailability of the tests, supportive investigations such as diaphragmatic electromyography or ultrasound fluoroscopy could not be performed. Based on history, clinical examination, and abdominal ultrasonographic findings of diaphragm myoclonus, along with excluding other causes, the diagnosis of BBD was made.

The patient was treated with oral and inhaled steroids for bronchial asthma, and salbutamol nebulization was stopped. Rapid intermittent movements that initially occurred every 3-5 min gradually spanned to every 2-3 hr on the day of admission. These movements eventually stopped around 14 hr after the discontinuation of salbutamol. During the first follow-up, the movements had subsided, and treatment for bronchial asthma continued. One week following follow-up, the child developed similar abdominal wall movements without using any medication. A neurologist also evaluated the child for pediatric autoimmune neuropsychiatric disorders associated with streptococcal infection, with antistreptolysin titer, throat swab, and blood culture. All these investigations were normal. In this second episode, the child was treated with promethazine and clonazepam tablets for a few days, although symptoms continued. Further inquiry with the caretaker revealed underlying psychological distress. Therefore, psychological counseling and treatment with Clonazepam were continued for two weeks, after which the symptoms subsided. The child has now been asymptomatic for two months.

## DISCUSSION

BDD is a rare condition first reported in 1990.^2^ Although described in various case reports, a reasonable pathophysiologic basis or possible mechanism for these disorders has yet to be described.^1^ Multiple terms have been used interchangeably to describe this phenomenon, such as belly dancer's syndrome, belly dancer's myoclonus, Leeuwenhoek's disease, respiratory myoclonus, moving umbilicus dyskinesia, moving umbilicus syndrome, truncal dystonia, abdominal dystonia, abdominal myoclonus, diaphragmatic tremor, and diaphragmatic myoclonus.^3^ These involuntary, infrequent movements usually occur on both sides of the abdomen. However, sometimes it can occur on one side of muscles like the diaphragm, rectus abdominis, oblique muscles, and paraspinal and perineal muscles. These movements can be aggravated with respiration and attenuated with sleep.^3,4^ In our patient, movements could be observed on both sides of the diaphragm, which disappeared during sleep.

Various causes have been documented in the literature, such as central nervous system disorders (organic and psychological disorders), peripheral nervous system disorders, pleural disorders, cardiac disorders, mediastinal disorders, cervical-spinal disorders, and drug-induced and idiopathic causes.^3^ Common drugs leading to BDD include dopamine-regulating medication such as L-dopa and antidopaminergic (quetiapine and prochlorperazine). Agents like domperidone, clebopride, and salbutamol have also been identified.^4-6^ Salbutamol was presumed as the initiating factor in this child as it was the first time he was nebulized with beta-adrenergic agonists to treat bronchial asthma. There was no history of any other drug intake apart from salbutamol. There is little known evidence of the association between salbutamol and myoclonus. It has been proposed that dose-dependent excessive stimulation of the catecholaminergic receptors of the central nervous system and skeletal muscles induces myoclonus.^7-9^ Although the salbutamol dosage used was unknown in our case, initiation of movement after the salbutamol nebulization indirectly points towards an exacerbation of myoclonus by salbutamol. Psychogenic factors such as hysteria and anxiety are significant causes of central nervous system disorders.^10^ In this patient, underlying psychogenic factors might have been the probable cause for the reoccurrence of the symptoms, which got better with counseling and clonazepam.

Diagnosis is usually based on the history and clinical features of the patient. History should include the current health status, drug use, and history of neurologic and psychogenic problems. The movements in BDD are occasionally painful and sometimes may present with chest pain, like in our patient. Other presenting symptoms may be shortness of breath, fatigue, palpitation, nausea, and vomiting.^3,4^ Investigations such as MRI head, echo, ECG, and chest x-ray can be done to rule out central and peripheral nervous system disorders, cardiac disorders, and pleural disorders. All these investigations were normal in this child.

Supportive investigations such as diaphragmatic electromyography or ultrasound fluoroscopy can be done to observe and confirm irregular diaphragmatic contractions. However, these tests could not be performed due to their unavailability. There is no examination finding to suggest an alternative diagnosis apart from the temporal association of salbutamol with BDD in our patient.

There are no clinical studies/trials to address the specific treatment for BDD; the suggested treatment is based on case reports and expert opinions. Drugs like diphenhydramine, benzodiazepine, levetiracetam, bupivacaine, and gabapentin have been used.^3,4^ Levetiracetam is more effective than benzodiazepine as it has fewer side effects. However, our patient was treated with a short course of clonazepam.^3^ When pharmacologic treatments are ineffective, phrenic nerve crush or block is also done in the older population; however, data on children is still lacking. Another new treatment modality is the injection of botulism toxin A under ultrasound guidance.^4^ Other methods have also been tried to treat BDD depending on the cause; thus, the prognosis is uncertain. In our case, salbutamol nebulization was the primary cause of BDD in a patient with an underlying psychological problem that subsided after medication and psychological counseling.

Belly dancer's dyskinesia is a disorder that is difficult to diagnose and even more complex to treat. Underlying psychological stressors may lead to this condition which can be aggravated with drugs such as beta-2 agonists. The exact pathophysiology or mechanism is not well understood. In some instances, like ours avoiding the drug, counseling, and medical therapy can control symptoms, while in other instances, surgical treatment may be required to ameliorate the disease.
